# Cardiorespiratory collapse at high temperature in swimming adult sockeye salmon

**DOI:** 10.1093/conphys/cot008

**Published:** 2013-05-24

**Authors:** Erika J. Eliason, Timothy D. Clark, Scott G. Hinch, Anthony P. Farrell

**Affiliations:** 1 Department of Zoology, University of British Columbia, Vancouver, Canada V6T 1Z4; 2 Faculty of Land and Food Systems, University of British Columbia, Vancouver, Canada V6T 1Z4; 3 Department of Forest Sciences, University of British Columbia, Vancouver, Canada V6T 1Z4

**Keywords:** Aerobic scope, cardiovascular, climate change, heart rate, migration, oxygen- and capacity-limited thermal tolerance

## Abstract

Elevated summer river temperatures are associated with high in-river mortality in adult sockeye salmon (*Oncorhynchus nerka*) during their once-in-a-lifetime spawning migration up the Fraser River (British Columbia, Canada). However, the mechanisms underlying the decrease in whole-animal performance and cardiorespiratory collapse above optimal temperatures for aerobic scope (*T*_opt_) remain elusive for aquatic ectotherms. This is in part because all the relevant cardiorespiratory variables have rarely been measured directly and simultaneously during exercise at supra-optimal temperatures. Using the oxygen- and capacity-limited thermal tolerance hypothesis as a framework, this study simultaneously and directly measured oxygen consumption rate (*M*O_2_), cardiac output , heart rate (*f*_H_), and cardiac stroke volume (*V*_s_), as well as arterial and venous blood oxygen status in adult sockeye salmon swimming at temperatures that bracketed *T*_opt_ to elucidate possible limitations in oxygen uptake into the blood or internal delivery through the oxygen cascade. Above *T*_opt_, the decline in *M*O_2max_ and aerobic scope was best explained by a cardiac limitation, triggered by reduced scope for *f*_H_. The highest test temperatures were characterized by a negative scope for *f*_H_, dramatic decreases in maximal  and maximal *V*_s_, and cardiac dysrhythmias. In contrast, arterial blood oxygen content and partial pressure were almost insensitive to supra-optimal temperature, suggesting that oxygen delivery to and uptake by the gill were not a limiting factor. We propose that the high-temperature-induced *en route* mortality in migrating sockeye salmon may be at least partly attributed to physiological limitations in aerobic performance due to cardiac collapse via insufficient scope for *f*_H_. Furthermore, this improved mechanistic understanding of cardiorespiratory collapse at high temperature is likely to have broader application to other salmonids and perhaps other aquatic ectotherms.

## Abstract (precis)

Elevated river temperatures have been associated with high mortality in migrating adult sockeye salmon, which may be partially attributed to physiological limitations in aerobic performance. This study suggests that the decrease in aerobic scope above optimum temperatures is due to a cardiac limitation, triggered by reduced scope for heart rate.

## Introduction

Temperature has been coined the ‘ecological master factor’ for ectotherms because of its influence on the biochemistry, physiology, behaviour, and ecology of animals (Fry, 1971). The optimal temperature (*T*_opt_) window for performance may be narrow in stenotherms, such as sockeye salmon (*Oncorhynchus nerka*), or broad in eurytherms, such as killifish (*Fundulus heteroclitus*; Eliason *et al.*, 2011; Healy and Schulte, 2012). Outside of the *T*_opt_ window, whole-animal performance declines until death eventually occurs. The *T*_opt_ for aerobic scope has been established as a useful metric for the successful return of adult sockeye salmon to their natal spawning areas in the Fraser River watershed (British Columbia, Canada; Farrell *et al.*, 2008). Indeed, *T*_opt_ appears to be locally adapted among sockeye salmon populations to the temperatures that they are likely to encounter once they enter the river (Eliason *et al.*, 2011). Peak summer river temperatures have increased by around 2°C since the 1950s (Patterson *et al.*, 2007), forcing salmon to migrate at temperatures warmer than their *T*_opt_ for aerobic scope. Biotelemetry studies have shown that high river temperature is correlated with excessive (>70%) mortality among migrating salmon (Crossin *et al.*, 2008; Farrell *et al.*, 2008; Mathes *et al.*, 2010; Martins *et al.*, 2011). Clearly, a conservation concern exists for future salmon migrations in this and other watersheds. As a result, there is great interest in the mechanism(s) underlying the decline of whole-animal performance and cardiorespiratory collapse above *T*_opt_.

Physiological processes are critical in defining thermal limits and temperature-induced mortality (Wang and Overgaard, 2007). Aerobic scope, defined as the difference between maximal oxygen consumption (*M*O_2max_) and resting oxygen consumption (*M*O_2rest_; Fry, 1947), represents the amount of oxygen available for activity. By definition, aerobic scope remains largely unchanged from its maximum within a defined *T*_opt_ window. However, at the critical temperature (*T*_crit_), when *M*O_2rest_ and *M*O_2max_ intersect, aerobic scope is zero and survival becomes passive, time limited, and supported by anaerobic metabolism (Pörtner, 2001; Pörtner and Farrell, 2008), a situation clearly incompatible with a highly aerobic upriver migration. Here we were specifically interested in which process triggers the decline in aerobic scope at a supra-optimal temperature. In the context of the present study, the term *T*_opt_ refers to the optimal temperature for aerobic scope.

While it is unclear what determines the limits of upper thermal tolerance in ectotherms, one leading possibility is the oxygen- and capacity-limited thermal tolerance (OCLTT) hypothesis, which attributes the decline in aerobic scope above *T*_opt_ to capacity limitations of the organ systems delivering oxygen to the tissues (Pörtner, 2001; Pörtner and Knust, 2007). Several studies with aquatic ectotherms have found support for the OCLTT hypothesis (e.g. fish and invertebrates; Frederich and Pörtner, 2000; Mark *et al.*, 2002; Lannig *et al.*, 2004; Pörtner and Knust, 2007; Pörtner and Farrell, 2008; Eliason *et al.*, 2011; Verberk and Calosi, 2012), while recent studies with pink salmon (*Oncorhynchus gorbuscha*), an intertidal snail (*Echinolittorina malaccana*), and an air-breathing toad (*Rhinella marina*) found little support for the OCLTT hypothesis (Clark *et al.*, 2011; Marshall *et al.*, 2011; Overgaard *et al.*, 2012). According to the OCLTT hypothesis, any one of the five major steps in the oxygen cascade from the environment to the mitochondria might trigger the collapse above *T*_opt_ (i.e. oxygen delivery by gill ventilation, oxygen diffusion across the gills, oxygen transport via the circulatory system, oxygen diffusion across tissue capillaries, and oxygen use by mitochondria; Weibel, 1984). However, simultaneous and preferably direct measurements of *M*O_2_, cardiac output 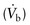, heart rate (*f*_H_), and cardiac stroke volume (*V*_s_), as well as arterial and venous blood oxygen status [partial pressure (*P*_O2_) and content (*C*_O2_)] are required to discover the initial limitation in the oxygen cascade that triggers the decline in aerobic scope above *T*_opt_. For example, a limitation in either oxygen delivery to or oxygen diffusion across the gill would be manifest by a decrease in arterial *P*_O2_ (*P*_aO2_) and arterial *C*_O2_ (*C*_aO2_). Alternatively, a cardiac limitation would be indicated by a failure of 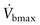 to increase to match the increased tissue oxygen demand above *T*_opt_. Finally, a limitation in oxygen diffusion to mitochondria in the working muscles would be indicated by venous *P*_O2_ (*P*_vO2_) remaining constant with increased tissue oxygen demand above *T*_opt_. However, support for a single limiting site in the oxygen cascade of fish (gills, heart, or locomotory muscle) remains incomplete despite considerable analysis (Brett, 1971; Heath and Hughes, 1973; Taylor *et al.*, 1996, 1997; Farrell, 2007a; Steinhausen *et al.*, 2008; Farrell *et al.*, 2009). This data gap exists because most studies examining temperature effects on aerobic scope in fish have not directly measured all the required variables (e.g. Fry, 1947; Brett, 1971; Taylor *et al.*, 1996), and the more comprehensive studies only used resting fish, thus failing to examine aerobic scope (e.g. Heath and Hughes, 1973; Cech *et al.*, 1976; Sartoris *et al.*, 2003; Gollock *et al.*, 2006; Clark *et al.*, 2008b; Keen and Gamperl, 2012). In fact, only Steinhausen *et al.* (2008) have directly measured all the required variables as a function of temperature in exercising fish.

The objective of this study was to examine the mechanism(s) that triggers the decline in *M*O_2max_ and aerobic scope above *T*_opt_ in sockeye salmon. Previous work suggested that sockeye salmon thermal limits are set by physiological limitations in aerobic performance, which may possibly be due to cardiac collapse at high temperatures (Steinhausen *et al.*, 2008; Eliason *et al.*, 2011). However, Eliason *et al.* (2011) did not systematically examine all the possible mechanisms leading to cardiorespiratory collapse (only aerobic scope, cardiac scope, scope for heart rate, *M*O_2rest_, and *M*O_2max_ were reported as a function of temperature), and Steinhausen *et al.* (2008) only swam sockeye salmon up to ∼75% of their critical swim speed (*U*_crit_). Thus, the present study reports the first simultaneous and direct measurements of all the pertinent oxygen transport variables in adult sockeye salmon swimming maximally below, at, and above *T*_opt._ Novel data on *M*O_2_, 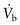, *f*_H_, and *V*_s_, as well as arterial and venous *P*_O2_, *C*_O2_, and blood chemistry are presented. We hypothesize that a limitation on *f*_H_ triggers cardiorespiratory collapse when temperature exceeds *T*_opt_ for aerobic scope.

## Materials and methods

### Fish collection

All procedures were approved by the University of British Columbia's Animal Care Committee (Animal use protocols A06-0328 and A08-0388) in accordance with guidelines recommended through the Canadian Council on Animal Care. Wild adult sockeye salmon (*n* = 55; body mass, 2380 ± 60 g; fork length, 59.6 ± 0.4 cm) were collected in 2007, 2008, and 2009 early in their upriver spawning migration (∼100 km upstream of the Fraser River mouth) and transported to the Fisheries and Oceans Canada Cultus Lake Research Laboratory (Cultus Lake, BC, Canada), where experiments were conducted. All fish were given a unique PIT (Passive Integrated Transponder; Biomark Inc., Boise, ID, USA) tag for individual identification, a scale was removed, and <0.1 g of the adipose fin was clipped for population identification via DNA analysis (Beacham *et al.*, 2005). Only fish from Early Stuart (*n* = 21), Chilko (*n* = 22), and Quensel (*n* = 12) populations were used in the present study. Fish were held at 11–12°C for 1–4 weeks in outdoor 8000–12 000 l circular aquaria under seasonal photoperiod.

### Surgical procedures

Individual fish were anaesthetized with buffered tricaine methanesulfonate in freshwater (0.2 g l^−1^ NaHCO_3_ and 0.1 g l^−1^ MS-222; Sigma, St Louis, MO, USA), weighed, and transferred onto wet foam on a surgical table, where their gills were continuously irrigated with aerated, chilled freshwater with a lower dose of buffered anaesthetic (0.15 g l^−1^ NaHCO_3_ and 0.075 g l^−1^ MS-222). Surgical procedures have been detailed elsewhere (Steinhausen *et al.*, 2008; Eliason *et al.*, 2011). Fish were instrumented with a 3 mm SB flowprobe (lateral cable exit; Transonic Systems, Ithaca, NY, USA) around the ventral aorta (Steffensen and Farrell, 1998) to measure 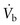, *f*_H_, and *V*_s_. Cannulae made of PE-50 tubing were inserted into the dorsal aorta (Soivio *et al.*, 1973) and sinus venosus (Farrell and Clutterham, 2003) to sample arterial and venous blood, respectively, for measurement of oxygen status and blood chemistry.

### Swim challenge to ***U***_**crit**_

Three days prior to the swimming test, most fish were placed in a 1400 l circular tank, and the temperature was progressively increased from the holding temperature (11–12°C) to the test temperature (12–22°C) by no more than 5°C day^−1^. The fish were maintained at their test temperature for 24–48 h before surgery was conducted. Following surgery, the fish were placed individually in one of two Brett-type swim tunnels (described by Lee *et al.*, 2003b; Steinhausen *et al.*, 2008) and allowed to recover overnight (>8 h) at their test temperature (12–22°C) at a low water velocity of ∼0.39 body lengths per second (bl s^−1^). Resting measurements of all variables were made following overnight recovery from surgery in the swim tunnel. Fish then underwent two sequential ramp-*U*_crit_ swim protocols with a 45  min recovery in between (Jain *et al.*, 1997; Lee *et al.*, 2003b; Eliason *et al.*, 2011). Only data from the first swim are used here.

Fish could not be held at the temperature extremes due to logistical constraints on temperature regulation in the holding tanks. Therefore, some fish recovered overnight (>8 h) from surgery in the swim tunnel at the holding temperature (12°C); initial resting values were measured at this temperature in the morning, and then the water temperature was increased or decreased by 4°C h^−1^ to the test temperature of either 8–10 or 22–26°C. One hour after reaching the test temperature, resting variables were recorded, and the fish underwent a single ramp-*U*_crit_ swim challenge.

The *M*O_2_ was measured during the second half of every 20 min velocity interval using an Oxyguard probe (Point Four Systems, Richmond, BC, Canada) attached to a Windaq box (Dataq Instruments, Akron, ON, USA) interfaced with Labview software (version 6.0; National Instruments, Austin, TX, USA). The 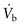 was measured continuously at 200 Hz throughout the swim trials by connecting the flowprobe to a flowmeter (Transonic Systems) interfaced with Biopac hardware and Acknowledge software (Biopac Systems, Santa Barbara, CA, USA). The value of 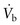 was calculated as the mean of three to six segments of continuous 30 s traces. Blood was strategically sampled prior to the swim test (rest), during steady-state swimming (steady), once the fish had transitioned to burst-and-coast swimming (burst), and immediately following the swim test within 5 min of fatigue (fatigue).

### Analysis of whole blood and plasma

Partial pressure of oxygen (*P*_O2_), oxygen content (*C*_O2_), haemoglobin concentration ([Hb]), and haematocrit (Hct) were measured using whole blood samples. Blood *P*_O2_ was measured using a blood gas monitor (PHM 73; Radiometer, Copenhagen, Denmark), which was calibrated and maintained at the experimental temperature using a water jacket. Blood *C*_O2_ was measured according to the method of Tucker (1967). The [Hb] was measured using either a handheld haemoglobin analyser (HemoCue 201^+^; Ängelholm, Sweden) calibrated for fish blood (Clark *et al.*, 2008a) or the spectrophotometer method with Drabkin's solution (Drabkin and Austin, 1935). Haematocrit was measured in duplicate using microhaematocrit capillary tubes spun at 10 000*g*. The remaining blood was centrifuged at 7000*g*, and the plasma was flash frozen in liquid nitrogen and stored at −80°C for subsequent analyses. Plasma glucose and lactate (YSI 2300 Stat Plus analyser), sodium and potassium (Cole-Parmer, model 41, single channel flame photometer) and chloride (Haake Buchler digital chloridometer) were measured using techniques outlined previously (Farrell *et al.*, 2001).

### Data analysis and statistics

The *U*_crit_ was calculated using established methods (Brett, 1964), after accounting for the solid blocking effect as outlined by Bell and Terhune (1970). Stroke volume (*V*_s_) was calculated as follows: 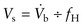. Cost of transport (COT) was calculated as follows: COT = *M*O_2_/(*U* × 60), where *U* is the swimming speed in metres per second. Net cost of transport (COT_net_) was calculated as follows: COT_net_ = (*M*O_2_ – *M*O_2rest_)/(*U* × 60). Likewise, cost of transport for cardiac output 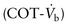 and net cost of transport for cardiac output 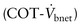 were calculated. Oxygen extraction (*A*–*V*_O2_) was calculated as follows: *A*–*V*_O2_ = *C*_aO2_ – *C*_vO2_. Arterial oxygen transport (*T*_aO2_) to the tissues was calculated as follows: 
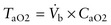
. Venous oxygen transport (*T*_vO2_) to the spongy myocardium and gills was calculated as follows: 
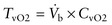
. Mean corpuscular haemoglobin concentration (MCHC) was calculated as follows: MCHC = [Hb]/(Hct/100).

In order to maximize statistical power, data were pooled for Early Stuart, Chilko, and Quesnel populations, because they undergo similar long, difficult river migrations, have a similar *T*_opt_ for aerobic scope (∼17°C) and have a similar maximal aerobic scope, cardiac scope and scope for heart rate (Eliason *et al.*, 2011). Moreover, Eliason *et al.* (under review) clearly show that *M*O_2_, 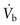, *f*_H_, and *V*_s_ for these three populations do not differ at rest, when swimming, or during recovery at *T*_opt_ for aerobic scope.

**Figure 1:**
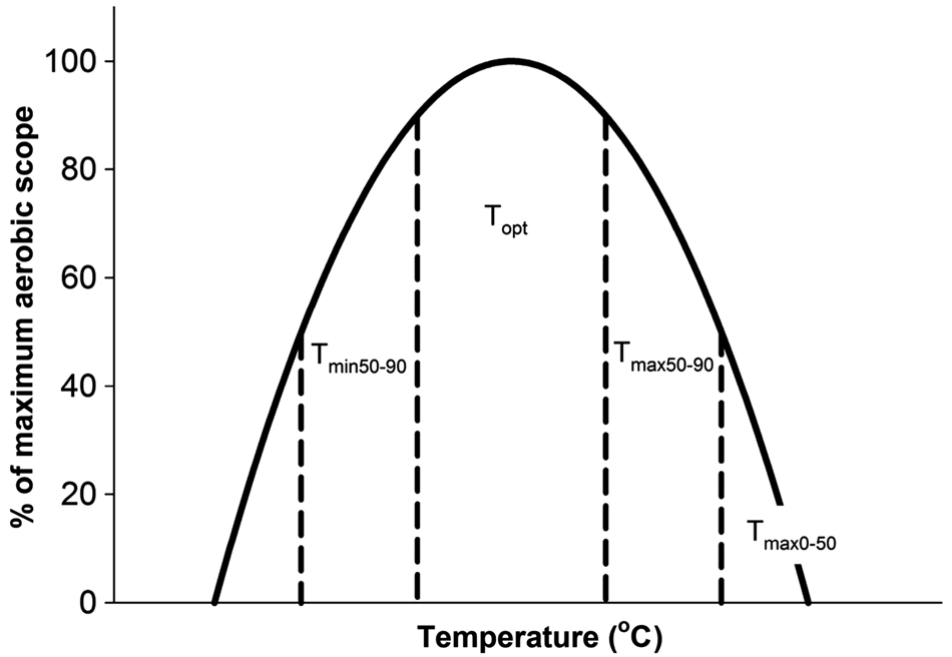
a schematic representation of the four temperature groupings used to analyse cardiorespiratory performance with temperature. The 50 and 90% cut-offs were assigned to individual fish based on their performance relative to *T*_opt_ for the population (see main text for details).

In order to permit statistical comparisons, individual fish were sorted into four temperature groupings based on performance relative to the maximal aerobic scope for a population (Fig. 1). The *T*_opt_ grouping (15–20°C; *n* = 28) combined individuals that attained 90–100% of the population-specific maximal aerobic scope. The assumption here is that the *T*_opt_ window is >90% of maximal aerobic scope. Bracketing the *T*_opt_ window were groupings of individuals that attained only 50–90% of the population-specific maximal aerobic scope. Thus, the grouping below *T*_opt_ was designated *T*_min50–90_ (*n* = 8; 12°C for Early Stuart, and 9–10°C for Chilko; no Quesnel fish were swum in this grouping), while the grouping above *T*_opt_ was designated *T*_max50–90_ (*n* = 11; 22–23°C for Early Stuart and Quesnel, and 24–25°C for Chilko). The *T*_max0–50_ grouping (*n* = 8) combined fish that attained 0–50% of the population-specific maximal aerobic scope at a supra-optimal temperature (23–26°C for Early Stuart and Quesnel, and 25–26°C for Chilko). In addition, a limited analysis was performed with the Chilko population alone to examine whether or not pooling data in the manner described above obscured or changed major cardiorespiratory trends.

Grouped data are presented as means ± SEM, unless otherwise indicated. Values of *P* < 0.05 were considered statistically significant. Independent data were compared using one-way ANOVA. Dependent data were compared using Student's paired *t*-test, a one-way repeated measures ANOVA or a two-way repeated measures ANOVA, as appropriate. When the requirement for normal distribution and equal variance could not be met after transformation, data were compared using appropriate non-parametric tests (e.g. Mann–Whitney *U*-test, Kolmogorov–Smirnov test, or Kruskal–Wallis test). The appropriate *post hoc* test (Holm–Sidak or Dunn's) was used to test for differences among groups. A polynomial quadratic equation was fitted to maximal and scope data for *M*O_2_, 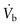, *V*_s_, and *f*_H_, and an exponential equation was fitted to the resting data for *M*O_2_, 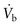, and *f*_H_ for individual Chilko sockeye salmon. A quadratic equation was fitted through the individual data for resting and fatigue venous *P*_O2_ and *C*_O2_.

## Results

### Cardiorespiratory and swimming performance

**Figure 2:**
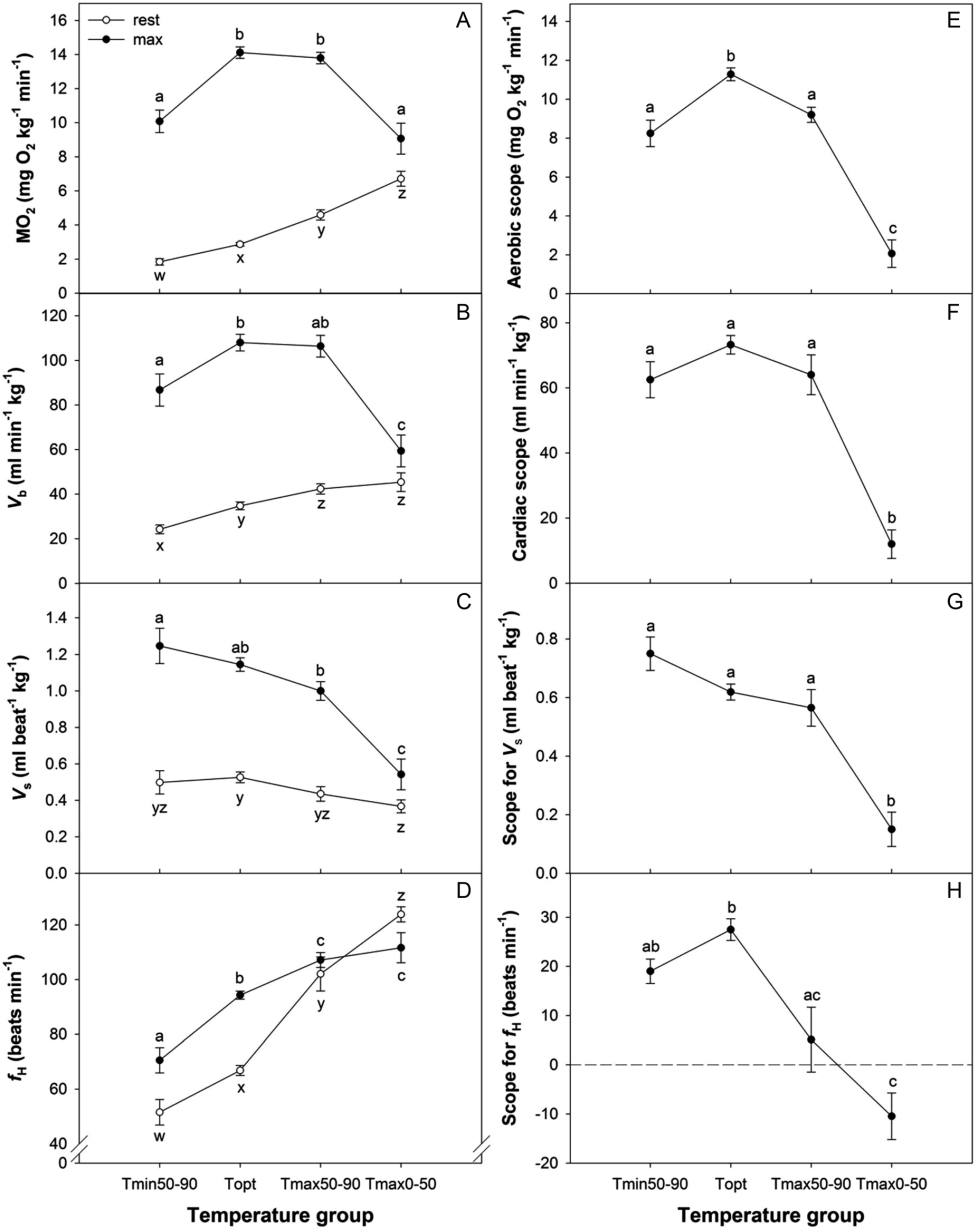
resting and maximal oxygen consumption rate (*M*O_2_; **A**), cardiac output (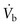; **B**), stroke volume (*V*_s_; **C**), and heart rate (*f*_H_; **D**) at the four temperature categories. Scope for *M*O_2_ (**E**), 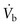 (**F**), *V*_s_ (**G**), and *f*_H_ (**H**) are shown. All values are presented as means ± SEM. Significant differences among temperature groupings are indicated by differing letters (*P* < 0.05).

**Figure 3:**
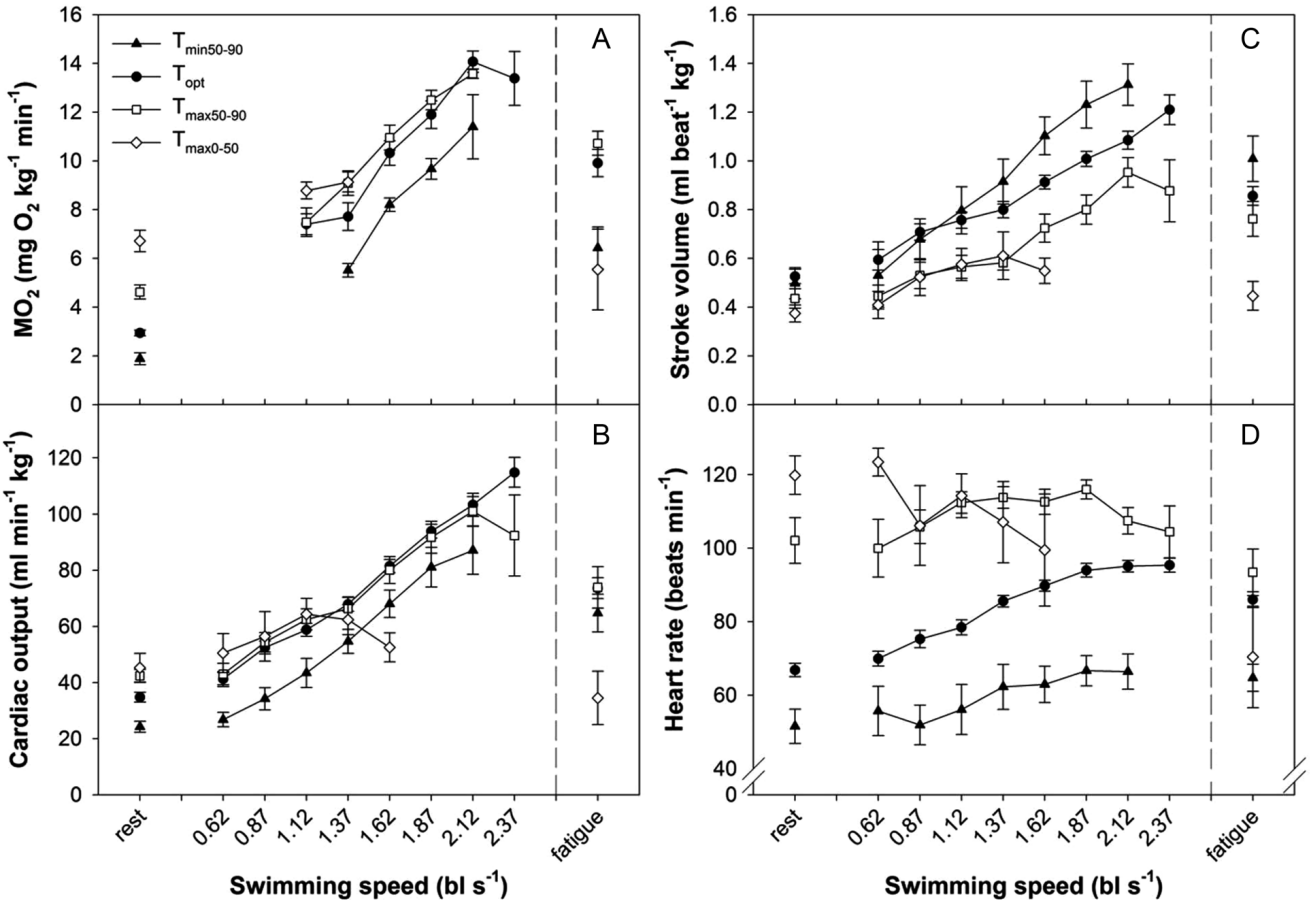
changes in oxygen consumption rate (**A**), cardiac output (**B**), cardiac stroke volume (**C**), and heart rate (**D**) as a function of swimming speed among the four temperature groupings. Fatigue values were obtained within 5 min after the fish stopped swimming. Means ± SEM are shown.

As intended from the grouping procedure, aerobic scope was highest for the *T*_opt_ grouping, significantly lower and similar for the *T*_min50–90_ and *T*_max50–90_ groupings, and lowest for *T*_max0–50_ grouping (Fig. 2E). Thus, the four performance groupings created the equivalent of a Fry curve for aerobic scope (Fry, 1947; Farrell, 2009). The Fry aerobic scope curve was a result of *M*O_2rest_ increasing progressively from the lowest to the highest temperature grouping (Fig. 2A), while *M*O_2max_ increased significantly between *T*_min50–90_ and *T*_opt_, was unchanged between *T*_opt_ and *T*_max50–90_, and then decreased significantly at *T*_max0–50_ (Fig. 2A). Indeed, swimming to fatigue significantly increasing *M*O_2max_ for the *T*_min50–90_, *T*_opt_, and *T*_max50–90_ groupings, while maximal swim speed and its corresponding *M*O_2max_ were significantly lower for the *T*_max0–50_ grouping compared with the other groupings (Fig. 3A).

Cardiac output varied among temperature groupings and with swimming, following similar patterns to *M*O_2_ (Figs 2 and 3). The resting cardiac output increased progressively with increasing temperature groupings, plateauing between *T*_max50–90_ and *T*_max0–50_, while increased significantly between *T*_min50–90_ and *T*_opt_, was unchanged between *T*_opt_ and *T*_max50–90_, and then decreased significantly at *T*_max0–50_. As a result, cardiac scope was lowest for the *T*_max0–50_ grouping (Fig. 2F).

Stroke volume at rest was independent of temperature except for a quantitatively small, but statistically significant decrease at *T*_max0–50_ when compared with *T*_opt_ (Fig. 2C). Scope for *V*_s_ was maintained between the *T*_min50–90_ and *T*_max50–90_ groupings, but decreased significantly for the *T*_max0–50_ grouping when *V*_smax_ also decreased significantly (Fig. 2C and G).

While *f*_Hrest_ increased progressively with each temperature grouping (Fig. 2D), *f*_Hmax_ increased with temperature only up to the *T*_max50–90_ grouping, plateauing between *T*_max50–90_ and *T*_max0–50_ (Fig. 2D). Therefore, scope for *f*_H_ was highest at *T*_opt_ (Fig. 2H). Notably, scope for *f*_H_ decreased precipitously at supra-optimal temperatures, approaching zero for the *T*_max50–90_ grouping, and becoming negative for the *T*_max0–50_ grouping (Fig. 2H).

**Figure 4:**
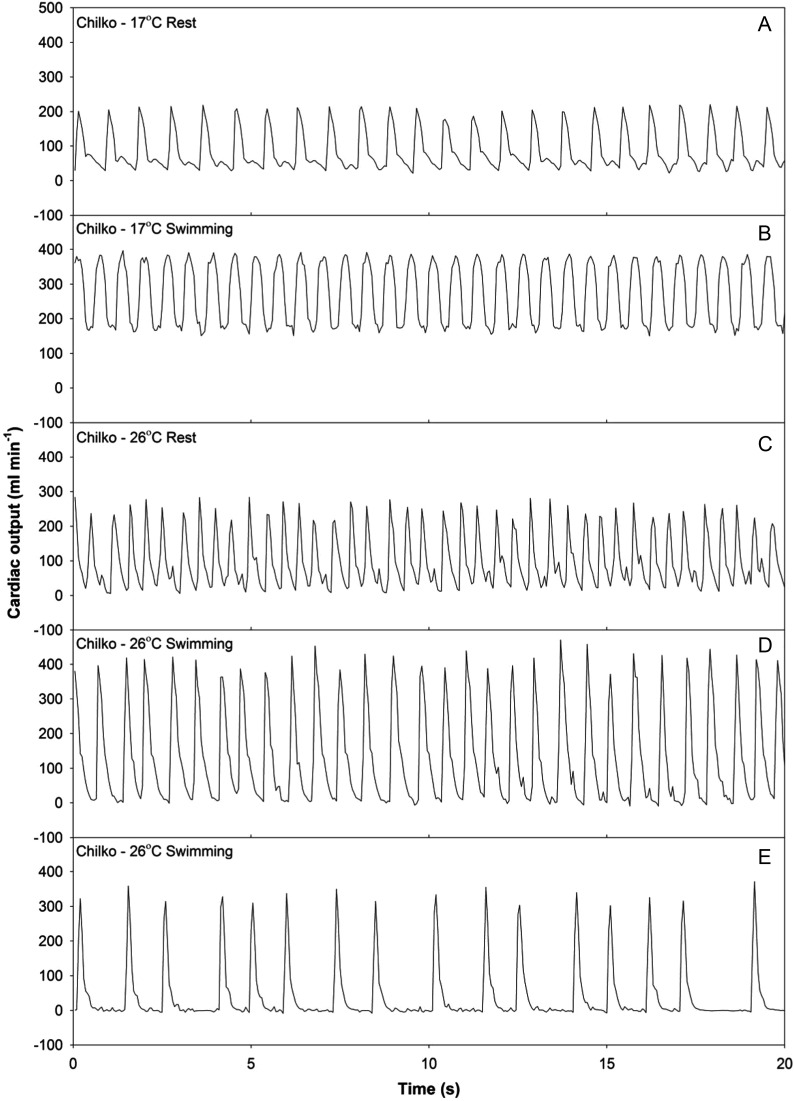
individual blood flow traces for two Chilko sockeye salmon at 17°C (*T*_opt_) and 26°C (*T*_max0–50_) at rest (**A** and **C**) and during swimming (**B**, **D**, and **E**). Swimming traces were recorded during the final swimming speed before each fish fatigued (measured at 2.3 and 1.5 bl s^−1^ for the 17 and 26°C fish, respectively). Trace D was recorded 5 min before trace E, at the same swimming speed.

In addition to the bradycardia during swimming and thus negative scope for *f*_H_, every fish that swam at *T*_max0–50_ exhibited an irregular heart beat immediately following the swim test, with 57% exhibiting cardiac dysrhythmias during the swim and shortly before fatigue (Fig. 4). Despite decreasing the temperature immediately following fatigue, 29% of the *T*_max0–50_ fish died, even though minutes before many were swimming at 1.1–1.5 bl s^−1^. Post-fatigue cardiac dysrhythmia was also present in the *T*_max50–90_ grouping, but at a lower percentage (27% of the fish), and none died post-exhaustion. Thus, the difference between post-fatigue life and death at supra-optimal temperature was a matter of 2–3°C. No cardiac dysrhythmias or deaths accompanied or followed swimming to exhaustion at either *T*_min50–90_ or *T*_opt_.

**Figure 5:**
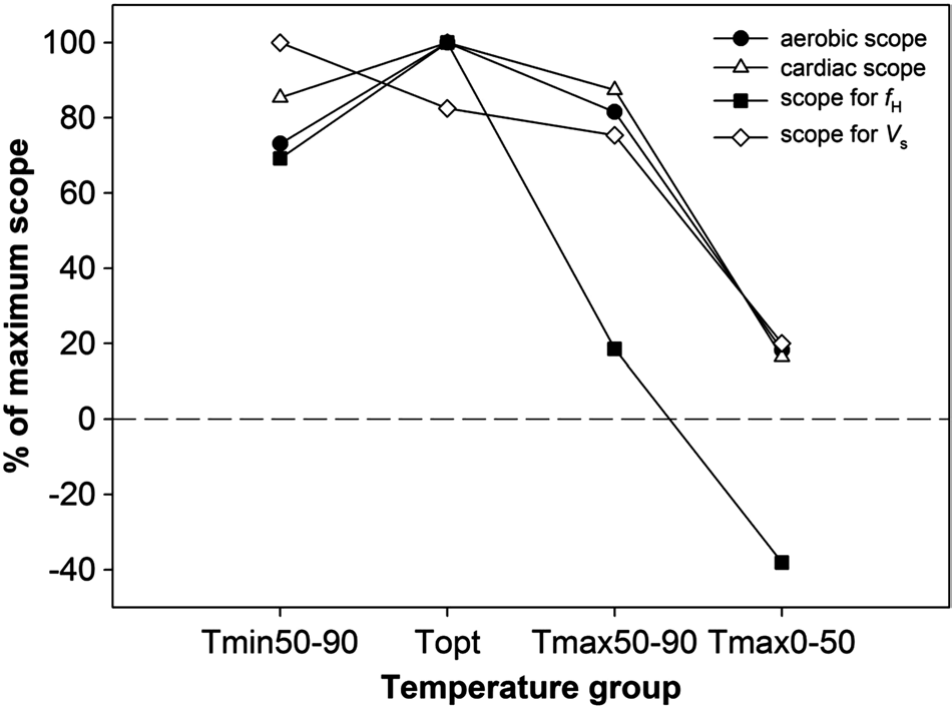
percentage of maximal aerobic scope, cardiac scope, scope for heart rate (*f*_H_), and scope for stroke volume (*V*_s_) for each temperature grouping.

Maximal scope occurred at *T*_opt_ for *M*O_2_, 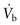, and *f*_H_, but at *T*_min50–90_ for *V*_s_ (Fig. 5). In comparison to these maximal values, scope for *M*O_2_, 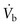, and *V*_s_ decreased by 13–25%, whereas scope for *f*_H_ plummeted by 80% for the *T*_max50–90_ grouping. Clearly, the precipitous collapse of scope for *f*_H_ was a potential trigger for the general cardiorespiratory collapse, which became clear and profound for the *T*_max0–50_ grouping, with scope for *M*O_2_, 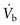, and *V*_s_ all declining to 16–20% of their maximal values, while scope for *f*_H_ collapsed to almost −40% of its maximal value (Fig. 5).

### Cardiorespiratory and swimming performance of Chilko sockeye salmon

**Figure 6:**
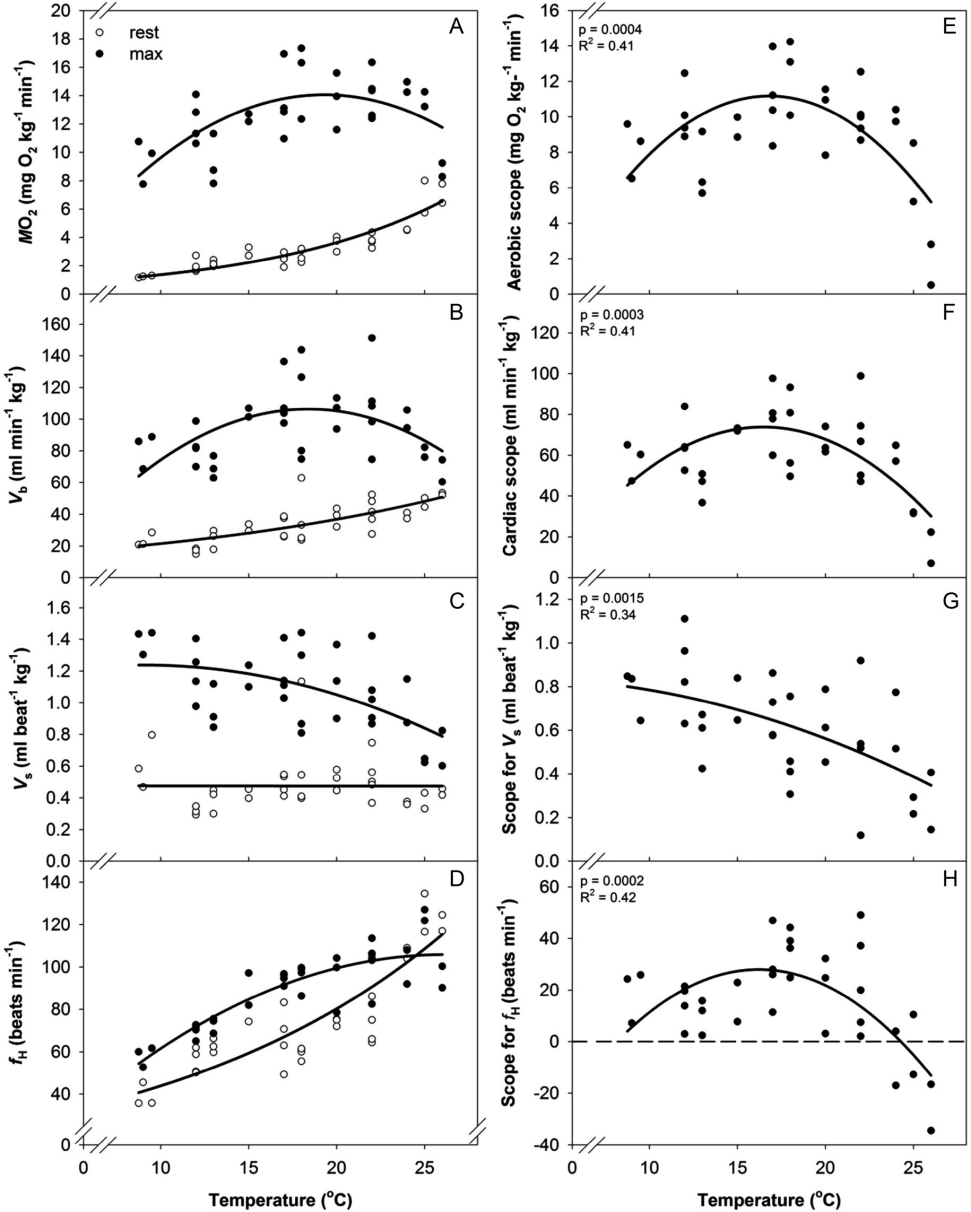
resting (open circles) and maximal values (filled circles) for oxygen consumption rate (*M*O_2_; **A**), cardiac output (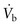; **B**), stroke volume (*V*_s_; **C**), and heart rate (*f*_H_; **D**) in Chilko sockeye salmon. Each point corresponds to a single fish. Scope for *M*O_2_ (**E**), 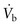 (**F**), *V*_s_ (**G**), and *f*_H_ (**H**) are shown. A polynomial quadratic equation was fitted to the maximum and scope data, an exponential equation was fitted to the resting data for *M*O_2_, 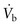, and *f*_H_, and no relationship was found with temperature for resting *V*_s_. Data in panels A and E and the equations for the lines for F and H have been previously reported by Eliason *et al.* (2011).

The pooling of populations accurately reflected the major cardiovascular trends seen for a single population, Chilko sockeye salmon swum at temperatures ranging from 8 to 26°C (Figs 2 and 6). Values of *M*O_2_, 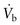, *V*_s_, and *f*_H_ all changed as a function of temperature in a similar manner to those of the pooled populations that were analysed statistically (Figs 2 and 6). Resting *M*O_2_, 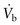, and *f*_H_ all increased exponentially with increasing temperature (Fig. 6), whereas *V*_srest_ was insensitive to temperature. Both *M*O_2max_ and 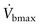 increased with increasing temperature until *T*_opt_ was reached, and both declined at the warmest temperatures. Maximal heart rate increased with increasing temperatures and plateaued above *T*_opt_, resulting in a negative scope for *f*_H_ at the warmest temperatures. Both *V*_smax_ and scope for *V*_s_ steadily declined with increasing temperatures (Fig. 6).

### Cost of swimming

**Figure 7:**
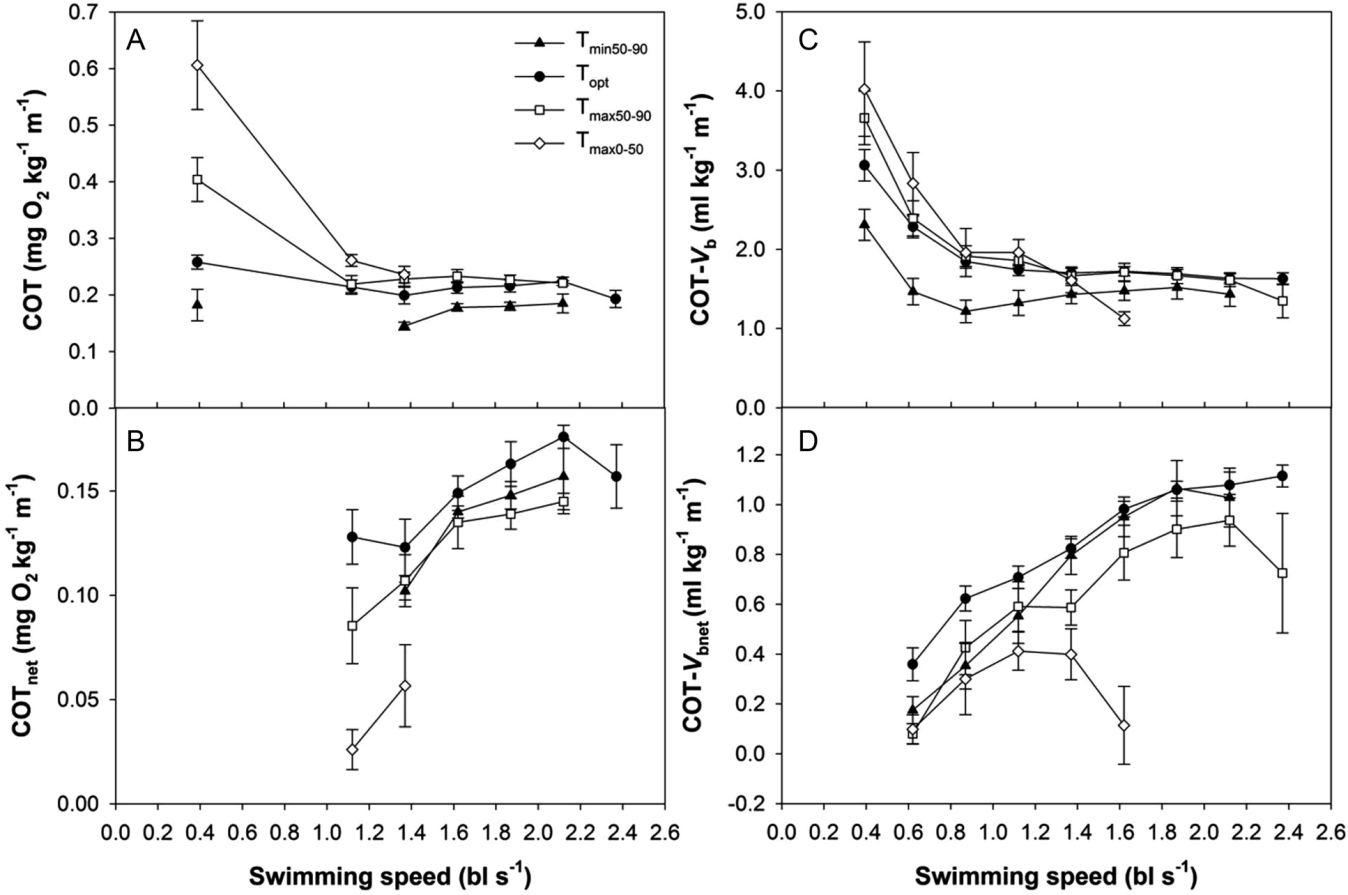
Cost of transport (COT; **A**), net cost of transport (COT_net_; **B**), cardiovascular cost of transport (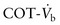; **C**), and net cardiovascular cost of transport (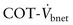; **D**) with swimming speed across the four temperature groupings. Means ± SEM are shown.

The oxygen cost of transport (COT) decreased with increasing swimming speed and reached a minimum of 0.12–0.26 mg O_2_ kg^−1^ m^−1^ across temperature groupings (Fig. 7). Temperature effects on COT were primarily evident at low speeds (0.4 bl s^−1^ = ‘rest’), with the warmer groupings (*T*_max50–90_ and *T*_max0–50_) tending to be twice as high compared with the colder groupings (*T*_opt_ and *T*_min50–90_). The pattern was reversed for the net cost of transport (COT_net_), which tended to be higher in the *T*_opt_ and *T*_min50–90_ groupings.

The circulatory cost of transport 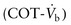 and net circulatory cost of transport for cardiac output 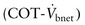 followed similar patterns with swimming and temperature to COT and COT_net_, respectively (Fig. 7). The circulatory cost of transport decreased with increasing swimming speeds until ∼0.87 bl s^−1^, after which it was maintained at around 0.7–1.9 ml kg^−1^ m^−1^ for the *T*_min50–90_, *T*_opt_, and *T*_max50–90_ groupings. In contrast, 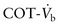 declined steadily in the *T*_max0–50_ grouping until fish stopped swimming. Again, 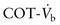 tended to be higher for warm temperature groupings; it was significantly lower at *T*_min50–90_ compared with the *T*_max50–90_ and *T*_max0–50_ groupings at the slowest speeds (Fig. 7); however, 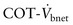 tended to be higher in the *T*_opt_ and *T*_min50–90_ groupings.

### Oxygen uptake, transport, and removal

**Figure 8:**
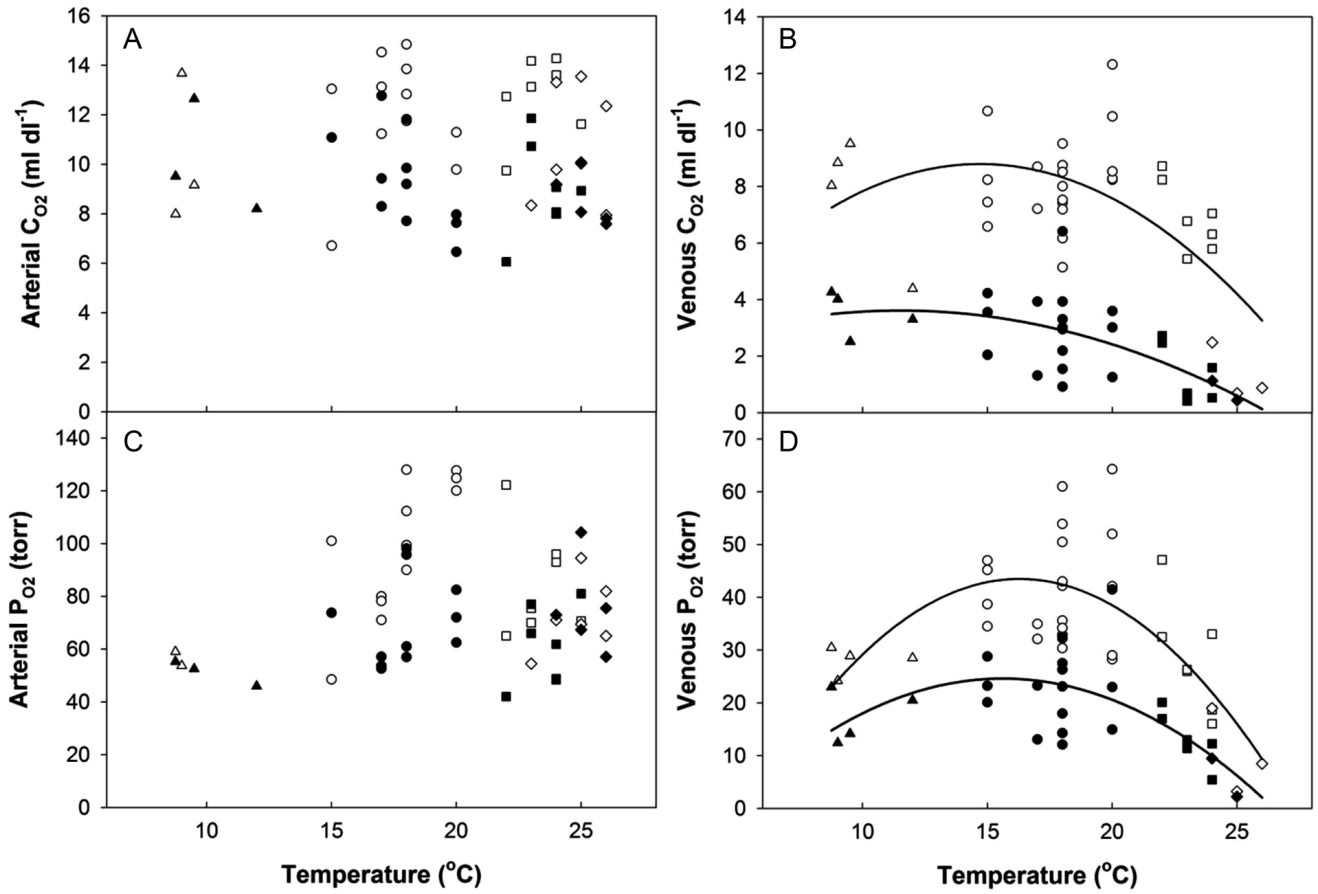
arterial and venous oxygen content (*C*_O2_; **A** and **B**, respectively) and partial pressure of oxygen (*P*_O2_; (**C** and **D**, respectively) at rest (open symbols) and fatigue (filled symbols) in four temperature groupings (triangles = *T*_min50–90_; circles = *T*_opt_; squares = *T*_max50–90_; and diamonds = *T*_max0–50_). Each data point corresponds to an individual fish. A quadratic equation was fitted through the venous data. For resting *C*_vO2_, *R*^2^ = 0.37, *P* = 0.0007; for fatigue *C*_vO2_, *R*^2^ = 0.39, *P* = 0.002; for resting *P*_vO2_, *R*^2^ = 0.51, *P* < 0.0001; and for fatigue *P*_vO2_, *R*^2^ = 0.41 *P* = 0.001.

The oxygen status of arterial blood was assessed from *P*_aO2_ and *C*_aO2_, which were found to be independent of temperature at all swimming speeds (Table 1, Fig. 8). In addition, resting *P*_aO2_ and *C*_aO2_ in the *T*_max50–90_ or *T*_max0–50_ groupings did not differ between the starting temperature (12°C) and the test temperature (22–26°C; Table 2). This result suggests that an acute temperature change while the fish were in the swim tunnel had no significant effect on arterial blood oxygen status. However, *P*_aO2_ and *C*_aO2_ tended to decrease relative to the resting values with swimming, as has been previously observed (Steinhausen *et al.*, 2008). Thus, oxygen transfer at the gills was unimpaired by supra-optimal temperature, even with the small effect of swimming on arterial oxygen status.

**Table 1:** oxygen status variables across the four temperature groupings and with swimming

	Rest	Steady	Burst	Fatigue
*P*_aO2_ (torr)				
*T*_min50–90_	–	60.7 ± 9.3	62.9 ± 3.5	49.8 ± 2.4
*T*_opt_	98.4 ± 7.4	96.5 ± 5.8	64.7 ± 9.6*	69.7 ± 4.9*
*T*_max50–90_	84.6 ± 7.7	71.9 ± 3.8	58.1 ± 4.3	60.7 ± 5.7
*T*_max0–50_	72.8 ± 4.8	70.8 ± 3.7	71.7 ± 7.4	75.4 ± 7.9
*P*_vO2_ (torr)				
*T*_min50–90_	28.0 ± 1.3^ab^	24.0 ± 1.1	–	17.5 ± 2.5
*T*_opt_	41.6 ± 2.4^a^	32.6 ± 1.9*	17.6 ± 2.8*	23.4 ± 2.0*
*T*_max50–90_	28.5 ± 3.9^b^	21.1 ± 3.3	11.6 ± 3.7*	13.2 ± 2.18*
*T*_max0–50_	10.3 ± 4.6^c^	–	–	–
*C*_aO2_ (ml dl^−1^)				
*T*_min50–90_	10.3 ± 1.7	11.6 ± 1.1	11.9 ± 1.2	11.6 ± 1.8
*T*_opt_	12.0 ± 0.6	10.9 ± 0.7	9.2 ± 0.8	9.3 ± 0.6
*T*_max50–90_	12.8 ± 0.6	11.2 ± 0.6	10.2 ± 0.5	9.0 ± 0.7*
*T*_max0–50_	10.8 ± 0.9	9.9 ± 1.6	9.2 ± 1.5	8.5 ± 0.5
*C*_vO2_ (ml dl^−1^)				
*T*_min50–90_	7.7 ± 1.1^a^	4.9 ± 1.2	–	3.5 ± 0.4^a^*
*T*_opt_	8.2 ± 0.4^a^	5.7 ± 0.4*	2.5 ± 0.3 *	3.0 ± 0.4^a^*
*T*_max50–90_	6.9 ± 0.5^a^	4.3 ± 0.8	1.0 ± 0.4*	1.4 ± 0.4^b^*
*T*_max0–50_	1.4 ± 0.6^b^	–	–	–
*A*–*V*_O2_ (ml dl^−1^)				
*T*_min50–90_	1.6 ± 1.6	–	–	6.8 ± 1.7*
*T*_opt_	4.2 ± 0.8	4.8 ± 0.5	6.3 ± 1.0	6.0 ± 0.7
*T*_max50–90_	6.1 ± 1.2	7.0 ± 0.9	9.2 ± 0.7	8.0 ± 1.4
*T*_max0–50_	–	–	–	–
*T*_aO2_ (ml O_2_ min^−1^ kg^−1^)				
*T*_min50–90_	3.4 ± 0.5	8.6 ± 1.0	13.4 ± 0.1^ab^*	11.1 ± 1.1^a^*
*T*_opt_	5.8 ± 0.4	9.4 ± 0.4*	14.7 ± 1.2^a^*	10.7 ± 0.8^a^*
*T*_max50–90_	7.6 ± 0.3	9.8 ± 0.4	13.7 ± 2.0^a^*	8.9 ± 1.7^ab^
*T*_max0–50_	7.2 ± 0.5	8.7 ± 1.2	7.4 ± 0.7^b^	4.1 ± 1.4^b^
*T*_vO2_ (ml O_2_ min^−1^ kg^−1^)				
*T*_min50–90_	2.8 ± 0.4^b^	3.8 ± 0.7		3.6 ± 0.4
*T*_opt_	4.1 ± 0.3^a^	5.1 ± 0.4	3.7 ± 0.4	3.0 ± 0.4
*T*_max50–90_	4.2 ± 0.4^ab^	3.8 ± 0.7	1.4 ± 0.5*	1.6 ± 0.5*
*T*_max0–50_	0.8 ± 0.6^b^	–	–	–

Arterial and venous partial pressures of oxygen (*P*_aO2_ and *P*_vO2_), oxygen content (*C*_aO2_ and *C*_vO2_), oxygen extraction (*A*–*V*_O2_), arterial oxygen transport (*T*_aO2_), and venous oxygen transport (*T*_vO2_) are indicated. Values are means ± SEM. Temperature groupings with different letters within a swimming speed are statistically different, and an asterisk indicates a statistically significant difference from rest within a temperature grouping (*P* < 0.05).

**Table 2:** arterial partial pressure of oxygen (P_aO2_) and oxygen content (C_aO2_) in resting fish, measured at 12°C and at the test temperature

	*n*	*P*_aO2_ (torr)		*C*_aO2_ (ml dl^−1^)	
		12°C	Test temp.	12°C	Test temp.
*T*_max50–90_	5	67.5 ± 3.3	81.0 ± 5.6	14.2 ± 0.6	13.4 ± 0.5
*T*_max0–50_	7	63.5 ± 6.4	72.8 ± 4.8	12.0 ± 0.4	10.8 ± 0.9

Means ± SEM are presented. There were no significant differences within a temperature group (*P* > 0.05).

Haemoglobin concentration, haematocrit, and mean corpuscular haemoglobin concentration were also independent of temperature and swimming speed, except that MCHC decreased at fatigue in the *T*_max50–90_ grouping relative to rest (Table 3). Given the stability of both *C*_aO2_ and [Hb] as a function of temperature, the changes in arterial oxygen transport (*T*_aO2_) as a function of temperature (and swimming speed) were primarily a result of changes in 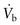. Notably, *T*_aO2_ for the *T*_max0–50_ grouping was significantly lower for burst swimming and at fatigue when compared with *T*_opt_ (Table 1). Indeed, while *T*_aO2_ increased during burst swimming by 294, 153, and 80% over the resting values for the *T*_min50–90_, *T*_opt_, and *T*_max50–90_ groupings, respectively, *T*_aO2_ was unchanged for the *T*_max0–50_ grouping (Table 1). Thus, the ability of the circulatory system to transport blood to active locomotory muscles was greatly diminished at supra-optimal temperatures because of a decline in 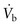, not *C*_aO2_.

**Table 3:** arterial haematological variables across the four temperature groupings and with swimming

	Rest	Steady	Burst	Fatigue
[Hb] (g l^−1^)				
*T*_min50–90_	92.3 ± 9.0	89.9 ± 10.6	93.5 ± 11.8	81.5 ± 12.6
*T*_opt_	92.4 ± 4.6	90.3 ± 4.2	91.8 ± 5.4	87.3 ± 3.0
*T*_max50–90_	100.3 ± 10.9	95.8 ± 2.7	89.3 ± 6.3	89.1 ± 6.3
*T*_max0–50_	89.0 ± 5.0	98.3 ± 6.0	91.6 ± 7.5	91.8 ± 2.3
Hct (%)				
*T*_min50–90_	30.7 ± 3.4	29.2 ± 1.6	30.6 ± 3.5	29.6 ± 5.2
*T*_opt_	31.0 ± 1.7	30.4 ± 1.6	33.9 ± 2.5	31.1 ± 1.4
*T*_max50–90_	32.9 ± 1.8	31.4 ± 0.8	34.2 ± 2.2	36.1 ± 1.4
*T*_max0–50_	32.6 ± 1.5	32.1 ± 0.9	35.8 ± 3.4	37.5 ± 3.3
MCHC (g l^−1^)				
*T*_min50–90_	302.2 ± 9.1	306.4 ± 19.7	305.1 ± 6.3	278.3 ± 10.9
*T*_opt_	300.5 ± 6.6	299.4 ± 6.8	273.1 ± 8.1	282.3 ± 4.9
*T*_max50–90_	308.4 ± 32.7	306.7 ± 13.5	267.7 ± 25.1	249.0 ± 22.0*
*T*_max0–50_	274.5 ± 15.8	305.7 ± 11.7	259.1 ± 19.4	250.4 ± 16.4
Glucose (mmol l^−1^)				
*T*_min50–90_	–	9.6 ± 0.7	9.0 ± 0.3	9.8 ± 0.7
*T*_opt_	5.6 ± 0.5	5.1 ± 0.5	6.6 ± 0.5	6.2 ± 0.6
*T*_max50–90_	7.6 ± 1.9	8.3 ± 1.2	6.9 ± 1.0	7.8 ± 1.1
*T*_max0–50_	10.2 ± 2.0	–	5.4 ± 1.9*	7.3 ± 1.6*
Lactate (mmol l^−1^)				
*T*_min50–90_	0.8 ± 0.4^a^	1.3 ± 0.3	2.3 ± 0.5	4.0 ± 0.9^a^
*T*_opt_	1.3 ± 0.2^a^	1.4 ± 0.2	3.5 ± 0.5	5.3 ± 0.6^ab^*
*T*_max50–90_	2.3 ± 0.5^ab^	2.5 ± 0.4	5.0 ± 0.6	9.5 ± 0.9^c^*
*T*_max0–50_	4.5 ± 0.7^b^	–	5.0 ± 1.4	8.0 ± 0.7^b^*
Na^+^ (mmol l^−1^)				
*T*_min50–90_	142.3 ± 2.9^a^	138.1 ± 1.1	133.1 ± 6.0	–
*T*_opt_	140.1 ± 1.7^a^	143.8 ± 1.6	145.9 ± 2.1	152.9 ± 2.0^a^*
*T*_max50–90_	120.7 ± 6.8^b^	140.4 ± 5.2*	138.9 ± 3.2*	139.6 ± 4.7^ab^*
*T*_max0–50_	137.7 ± 4.1^ab^	–	135.9 ± 2.6	137.6 ± 2.6^b^
K^+^ (mmol l^−1^)				
*T*_min50–90_	3.3 ± 0.1	3.9 ± 0.5	4.3 ± 0.8	4.0 ± 1.8
*T*_opt_	4.9 ± 0.4	4.6 ± 0.4	2.7 ± 0.7	2.6 ± 0.3*
*T*_max50–90_	6.2 ± 1.0	4.5 ± 0.7	2.8 ± 0.6*	1.7 ± 0.3*
*T*_max0–50_	3.8 ± 0.3	–	3.9 ± 0.9	1.7 ± 0.6
Cl^−^ (mmol l^−1^)				
*T*_min50–90_	121.0 ± 2.0^ab^	122.0 ± 5.2	117.4 ± 2.3^ab^	123.6 ± 3.8^ab^
*T*_opt_	127.5 ± 0.9^a^	129.9 ± 1.4	130.2 ± 1.3^a^	133.3 ± 1.2^a^
*T*_max50–90_	119.3 ± 6.0^ab^	119.8 ± 2.1	118.3 ± 4.5^ab^	119.9 ± 5.2^b^
*T*_max0–50_	112.0 ± 3.1^b^	–	113.4 ± 3.9^b^	111.9 ± 2.7^b^

Haemoglobin concentration ([Hb]), haematocrit (Hct), mean cell haemoglobin concentration (MCHC), plasma sodium (Na^+^), plasma potassium (K^+^), and plasma chloride (Cl^−^) are indicated. Values are means ± SEM. Temperature groupings with different letters within a swimming speed are statistically different, and an asterisk indicates a statically significant difference from rest within a temperature grouping (*P* < 0.05).

Unlike arterial blood, *P*_vO2_ and *C*_vO2_ both varied significantly with temperature and swimming (Table 1). The changes in *C*_vO2_ directly reflected changes in haemoglobin saturation, because [Hb] was unchanged (Table 3). The effect of temperature on resting *P*_vO2_ and *C*_vO2_ was profound. Indeed, resting *P*_vO2_ for the *T*_max0–50_ grouping was 10 torr, 25% of the *T*_opt_ value, and resting *C*_vO2_ was only 16–20% of the *C*_vO2_ for the other three temperature groupings. When temperature was treated as a continuous independent variable, the significant polynomial relationships also revealed decreases in resting *P*_vO2_ and *C*_vO2_ at the highest temperature (Fig. 8).

Swimming increased muscle oxygen extraction from the blood, as reflected by decreases of *P*_vO2_ and *C*_vO2_ (Table 1). At fatigue, *C*_vO2_ and *P*_vO2_ were 114 and 77%, respectively, lower in the *T*_max50–90_ grouping compared with *T*_opt_. Likewise, the significant polynomial relationship with temperature for both *P*_vO2_ and *C*_vO2_ at fatigue further illustrates the point that tissue oxygen extraction must have increased above *T*_opt_ (Fig. 8). Tissue oxygen extraction (*A*–*V*_O2_) was calculated only for individuals with paired, simultaneous arterial and venous samples. The tendency for *A*–*V*_O2_ to increase with swimming and at warmer temperatures did not reach statistical significance, probably due to low statistical power (Table 1).

Even though the tissues extracted more oxygen from the blood with swimming, the amount of oxygen leaving the tissues and returning to the heart (venous oxygen transport; *T*_vO2_) was independent of swimming effort at *T*_opt_ (Table 1). Thus, the increase in 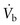 matched the decrease in *C*_vO2_. However, this situation changed at supra-optimal temperatures, because *T*_vO2_ significantly decreased at burst and fatigue for the *T*_max50–90_ grouping (Table 1). Even at rest, *T*_vO2_ was reduced by over 4-fold in the *T*_max0–50_ grouping compared with *T*_opt_ (Table 1).

### Other blood variables

Plasma lactate varied significantly with both temperature and swimming (Table 3). The effect of supra-optimal temperatures on plasma lactate was profound, because resting plasma lactate was >3-fold higher in the *T*_max0–50_ grouping compared with *T*_opt_ (Table 3). This result suggests that oxygen supply was insufficient to meet tissue oxygen demand even at rest for the highest test temperature. As expected, burst swimming to fatigue increased plasma lactate by 2- to 4-fold relative to resting levels for all temperature groupings. Moreover, plasma lactate was highest in fish at *T*_max50–90_ at fatigue compared with the other groups (Table 3). Plasma glucose was independent of temperature and swimming, except for a significant decrease in plasma glucose with swimming for the *T*_max0–50_ grouping (Table 3), perhaps reflecting a greater drain on this fuel for anaerobic metabolism.

Plasma sodium varied significantly with both temperature and swimming speed (Table 3), tending to be highest for the *T*_opt_ grouping and increasing with swimming speed for the *T*_opt_ and *T*_max50–90_ groupings. Plasma chloride also varied significantly with temperature, but not with swimming speed (Table 3), and was significantly higher at *T*_opt_ relative to the *T*_max0–50_ grouping. Plasma potassium did not vary significantly with temperature (Table 3), but tended to decrease with swimming speed, and was lowest at fatigue.

## Discussion

In order to apply physiological knowledge to conservation problems, scientists require a mechanistic understanding and the support of guiding principles. The present study sought to make such a contribution by examining the mechanism of the decline in *M*O_2max_ and aerobic scope above *T*_opt_ in sockeye salmon using the OCLTT hypothesis as a framework. Earlier temperature studies with fishes either did not measure all the required variables (i.e. *M*O_2_, 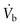, *C*_aO2_, and *C*_vO2_) or the fish were not swum maximally, if at all. For example, many studies have examined the collapse of cardiorespiratory performance during warming of resting fish (Heath and Hughes, 1973; Cech *et al.*, 1976; Sartoris *et al.*, 2003; Gollock *et al.*, 2006; Clark *et al.*, 2008b; Keen and Gamperl, 2012), but their results relate to what is triggering events close to *T*_crit_ as opposed to the collapse of aerobic scope above *T*_opt_.

Brett (1971) first described a parallel association of swimming performance, aerobic scope, and cardiac scope as a function of temperature between 5 and 22°C. He noted a decrease in aerobic scope, cardiac scope, and scope for heart rate above a *T*_opt_ of 15°C. However, was calculated using the Fick principle, and Brett concluded that the cardiorespiratory collapse above *T*_opt_ was likely to be due to limited oxygen availability, even though blood gases were not reported (Brett, 1964, 1971). Taylor *et al.* (1996) examined aerobic swimming of rainbow trout at three acclimation temperatures (4, 11, and 18°C), and indirectly estimated 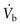 using microspheres. By coupling these cardiac measurements with an earlier study of blood gases (Taylor *et al.*, 1993), they concluded that aerobic swimming performance may have been limited at high temperature because of numerous factors, including reduced oxygen solubility in water and plasma, decreased arterial blood oxygen content and haematocrit, elevated energy expenditure for reproduction, reduced aerobic muscle mass or decreased cardiac scope, and reduced blood flow to swimming muscles. Steinhausen *et al.* (2008) was the first to measure *M*O_2_, 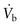, *C*_aO2_, and *C*_vO2_ simultaneously and directly in sockeye salmon as they were progressively warmed from 15 to 24°C while swimming steadily at ∼1.35 bl s^−1^ or ∼75% of maximum. They concluded that a cardiac limitation developed at warm temperatures in swimming fish, because 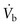, *f*_H_, and *T*_aO2_ reached maxima and failed to continue to increase with increasing temperatures in order to meet the increased tissue oxygen demand.

The present study is the most comprehensive assessment to date of aerobic scope and the associated cardiovascular and blood gas variables across a range of temperatures in an aquatic ectotherm. We conclude that a limitation in maximal cardiac performance inhibits *M*O_2max_ and aerobic scope at supra-optimal temperatures in salmonids, which is apparently triggered by a precipitous decrease in scope for *f*_H_. Thus, the present study with sockeye salmon found support for the OCLTT hypothesis, though we caution that the OCLTT hypothesis may not be broadly applicable to all ectotherms [e.g. pink salmon (Clark *et al.*, 2011), intertidal snails (Marshall *et al.*, 2011)*,* and air-breathing toads (Overgaard *et al.*, 2012)] and warrants further investigation. Of particular conservation concern for sockeye salmon is the additional finding that post-fatigue mortality occurred at temperatures only 3–5°C above *T*_opt_.

### Swimming performance

Salmonids are known to increase their reliance on anaerobic swimming in *U*_crit_ tests at supra-optimal temperatures (Brett, 1964; Jain and Farrell, 2003), a finding that lends support to the OCLTT hypothesis. This was clearly evidenced here by the elevated lactate levels at fatigue in the warmest temperature grouping, supporting the idea that oxygen delivery to tissues in swimming fish becomes limited at temperatures near the critical maximum. A novel finding in the present study was the elevated plasma lactate even at rest and the depletion of plasma glucose with swimming in the highest temperature grouping, providing further indications that tissue oxygen delivery was compromised. The difficulty of swimming at supra-optimal temperature was evident from the increased COT and 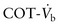 with temperature, indicating higher oxygen and cardiovascular costs to swimming at a given speed compared with cooler temperatures. In fact, the true COT and 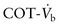 may be even higher, because the metabolic costs of anaerobic swimming require the characterization of excess post-exhaustion oxygen consumption (see Lee *et al.*, 2003a), which was not done here.

### Gill oxygen delivery and uptake

Water oxygen content decreases by around 2% °C^−1^ with increasing water temperature (Dejours, 1975), which limits environmental oxygen availability. Furthermore, the affinity of haemoglobin for oxygen decreases with increasing temperature (Perry and Reid, 1994; Jensen *et al.*, 1998; Clark *et al.*, 2008b), which may hamper oxygen uptake at the gill and potentially prevent full haemoglobin saturation. Therefore, strong theoretical arguments exist for a limitation in oxygen uptake at the gills to explain the collapse of aerobic scope above *T*_opt_ (through either insufficient water delivery to the gills or reduced diffusion of oxygen across the gills). If this were the case, *P*_aO2_ and *C*_aO2_ would decrease and potentially reduce *T*_aO2_ at temperatures above *T*_opt_.

There is considerable evidence for such changes in arterial oxygen status in resting, but not in exercising fishes. Keen and Gamperl (2012) showed a reduction in *C*_aO2_ and *P*_aO2_ by ∼25 and 35%, respectively, in resting steelhead trout warmed from 12°C to their critical thermal maximum. However, they noted that a limited capacity for ram ventilation in the test apparatus could have been a contributing factor. Clark *et al.* (2008b) found that resting adult Chinook salmon slightly decreased *C*_aO2_ and *P*_aO2_ when acutely warmed, but this was more pronounced in larger individuals. Heath and Hughes (1973) showed a large and parallel decrease in *C*_aO2_ and *C*_vO2_ with acute heat stress up to 25°C in resting rainbow trout, but did not measure Hct or [Hb] to check for haemodilution with repetitive blood sampling. The only report of *C*_aO2_ becoming lower during swimming was for seasonally acclimatized rainbow trout at 18°C when compared with 4 and 11°C, but haematocrit was also halved at 18°C (Taylor *et al.*, 1993).

In contrast to these works, both the present study and that of Steinhausen *et al.* (2008) found that *C*_aO2_, *P*_aO2_, and Hct were maintained independent of temperature in swimming adult sockeye salmon. Likewise, Sartoris *et al.* (2003) found that *P*_aO2_ remained constant during acute warming in resting Atlantic cod. A telling discovery here was that *C*_aO2_ was maintained even when cardiac dyssynchrony and post-fatigue delayed mortality occurred at extremely warm temperatures. As such, the present study clearly provides no support for an oxygen limitation developing at the gills at warm temperatures.

Given the different conclusions that have been reached for the effects of temperature on oxygen delivery and uptake at the gill, future studies must pay close attention to the response of Hct and [Hb] to temperature, which directly affects *C*_aO2_. Haematocrit has been shown to increase by up to 27% due to splenic contraction in acutely warmed resting rainbow trout (Sandblom and Axelsson, 2007), to decrease by 50% in warm-acclimated rainbow trout (Taylor *et al.*, 1993), and to be minimally affected by temperature (present study and see Farrell, 1997; Clark *et al.*, 2008b; Steinhausen *et al.*, 2008). Future studies must consider haemodilution, because repeated blood sampling can reduce Hct, [Hb], and *C*_aO2_, though this was not a problem in the present study. In addition, future studies should carefully consider the role of water velocity in assisting gill ventilation (ram ventilation), as was possible here in the swim tunnels, and the possibility that fish holding devices may restrict buccal and opercular movements during gill ventilation.

### Circulatory transport of oxygen

All studies that have measured 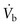 (directly or indirectly) and *M*O_2_ as a function of temperature in swimming salmonids have reported parallel declines in 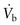 and *M*O_2_ above *T*_opt_ (Brett, 1971; Taylor *et al.*, 1996; Steinhausen *et al.*, 2008; Eliason *et al.*, 2011). Here we present additional evidence in support of a cardiac limitation developing at supra-optimal temperatures, because maximal *T*_aO2_, *M*O_2_, and 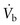 all failed to increase above *T*_opt_. In addition, all three variables decreased at *T*_max0–50_, at which point scope for *f*_H_ completely collapsed without any compensation by *V*_s_. Therefore, we conclude that a perfusion limitation develops above *T*_opt_, resulting in insufficient oxygen delivery to meet the increased locomotory tissue oxygen demand. Notably, scope for *f*_H_ collapsed at a lower temperature compared with scope for 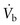, aerobic scope, and scope for *V*_s_ (Fig. 5). Therefore, we propose that reduced scope for *f*_H_ is the triggering mechanism that limits 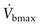 above *T*_opt_, supporting an earlier proposal (Farrell, 2009). It is becoming clear that *f*_H_ is a central mechanism setting upper temperature tolerance and thus may be a key limiting factor regulating fish distribution globally.

Changes in 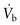 and *T*_aO2_ as a function of temperature are almost entirely mediated by changes in *f*_H_ in both resting and swimming fish (present study and Cech *et al.*, 1976; Gollock *et al.*, 2006; Sandblom and Axelsson, 2007; Clark *et al.*, 2008b, 2011; Steinhausen *et al.*, 2008), underscoring the central importance of *f*_H_ in determining cardiac performance and oxygen delivery with temperature. More broadly, the central importance of resting *f*_H_ in setting temperature tolerances for intertidal invertebrates is also gaining support (Somero, 2011). Direct effects of temperature on the cardiac pacemaker rate (Randall, 1970) are likely to account for temperature-induced cardiac acceleration. Here, resting *f*_H_ continued to increase until lethal temperatures were approached in the highest temperature group (range, 117–135 beats min^−1^; mean, 123.9 beats min^−1^). Moreover, *f*_Hmax_ plateaued above *T*_opt_, decreasing from rest at the highest test temperatures. Therefore, we suggest that factors that (i) determine *f*_Hrest_ and (ii) limit *f*_Hmax_ may be of particular importance for future studies.

The bradycardia followed by dysrhythmia in fish swimming at the highest temperature is a novel observation that adds to the observation of irregular heart beats in resting rainbow trout, Atlantic cod, and Chinook salmon acutely warmed to their *T*_crit_ (Heath and Hughes, 1973; Gollock *et al.*, 2006; Clark *et al.*, 2008b). Here, severe dysrhythmias were associated with dramatic decreases in *P*_vO2_, *C*_vO2_, and *T*_vO2_, although these sockeye salmon displayed an impressive tenacity to continue swimming, albeit at lower speeds and with a lower *M*O_2_, 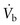, and *T*_aO2_. Such tenacity, however, may have severe consequences, because there was significant post-fatigue mortality shortly after exhaustion despite cooling the salmon. At temperatures a few degrees cooler, there was no such immediate consequence. However, we cannot rule out the possibility of delayed mortality, because it can occur hours to days after exhaustive exercise, both in the laboratory (e.g. Black, 1957; Wood *et al.*, 1983) and in nature (e.g. Donaldson *et al.*, 2012), especially at elevated temperature.

The mechanisms limiting maximal *f*_H_ and triggering bradycardia and cardiac dysrhythmia remain unclear. Maximal *f*_H_ is clearly unable to continue increasing with a *Q*_10_ of ∼2 at temperatures above *T*_opt_. The initial bradycardia during swimming may have been vagally mediated and could act as a protective mechanism by (i) reducing 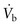 and cardiac oxygen demand (Farrell and Steffensen, 1987), (ii) increasing blood residence time in the heart to favour oxygen extraction by spongy myocardium (Farrell, 2007b), and (iii) reducing coronary vascular compression to favour a more continuous coronary blood flow to compact myocardium (Axelsson and Farrell, 1993; Gamperl *et al.*, 1995). The possibility of a centrally mediated bradycardia should be examined using atropine injection or vagotomy. Alternatively, synergistically or perhaps secondarily, the deleterious venous blood environment associated with anaerobic swimming at high temperature could have triggered cardiac problems, including the observed decrease in *f*_Hmax_ and *V*_smax_. Specifically, anaerobic swimming makes venous blood acidotic (low pH), hypoxaemic (low *P*_vO2_) and hyperkalaemic (high K^+^; Kiceniuk and Jones, 1977; Holk and Lykkeboe, 1998), all of which become exacerbated at high temperature (Brett, 1964; Jain and Farrell, 2003; Steinhausen *et al.*, 2008). Notably, hyperkalaemia was absent in the present study. These extracellular changes can impair cardiac contractility (Driedzic and Gesser, 1994), and even more so at high temperature (Hanson and Farrell, 2007). At extremely high temperatures, the decreases in *P*_vO2_ and *T*_vO2_ at warm temperatures may not have guaranteed sufficient oxygen supply to the avascular spongy myocardium (Farrell and Clutterham, 2003; Farrell, 2007a), resulting in reduced contractility and perhaps dysrhythmias. In fact, the lowest *P*_vO2_ observed at the highest temperature (10 torr) is around the suggested lower limit for adequate oxygen delivery to the fish spongy myocardium (Davie and Farrell, 1991). In contrast, the compact myocardium (supplied with oxygen by the coronary circulation) probably would not have suffered in a similar manner, because *C*_aO2_ and *P*_aO2_ were maintained. All told, the exact mechanisms and sequence of events leading to the *f*_H_ limitation are unclear and warrant further study.

Why resting fish do not increase *V*_s_ with acute warming remains unknown (present study and Cech *et al.*, 1976; Brodeur *et al.*, 2001; Gollock *et al.*, 2006; Sandblom and Axelsson, 2007; Clark *et al.*, 2008b; Steinhausen *et al.*, 2008; Mendonça and Gamperl, 2010; Gamperl *et al.*, 2011). Elevated temperature, *per se*, does not prevent an increase in resting *V*_s_, because zatebradine treatment, which reduces *f*_H_ at the level of the pacemaker, triggered a compensatory increase in *V*_s_ in resting rainbow and steelhead trout at high temperature (Gamperl *et al.*, 2011; Keen and Gamperl, 2012). All the same, supra-optimal temperatures can reduce *V*_smax_ in swimming salmon (present study and Clark *et al.*, 2011), something that was not revealed when sockeye salmon were swimming at ∼75% of *U*_crit_ (Steinhausen *et al.*, 2008). The mechanism of the decline in *V*_smax_ at extremely warm temperatures is unknown, but may be related to the deleterious venous blood environment associated with anaerobic swimming at high temperature, as outlined above.

### Tissue oxygen extraction

The elevation in plasma lactate in both resting and swimming fish is a clear indication that the increased tissue oxygen demand was not being met by oxygen delivery at supra-optimal temperature. This mismatch between oxygen supply and demand could have resulted from a perfusion and/or a diffusion limitation to the locomotory muscles. The present study clearly showed that arterial oxygen transport was compromised above *T*_opt_ due to reduced 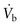, leading to a muscle capillary perfusion limitation. However, insufficient tissue oxygen extraction could also lead to increased anaerobic metabolism if a diffusion limitation for oxygen existed at the locomotory muscles. Factors leading to a diffusion limitation are inadequate capillary density, ineffective muscle cellular morphology (e.g. poor mitochondria density or location), and an insufficient driving force for oxygen diffusion (low *P*_aO2_; Egginton and Cordiner, 1997; Taylor *et al.*, 1997; Egginton, 2002). Thus, a diffusion limitation for oxygen is more likely for white rather than red muscle given the attendant ∼10 times lower capillary density of white compared with red muscle in salmonids (Mosse, 1978; Egginton and Sidell, 1989). However, none of these morphometric features was studied here, and so assessing the role of a diffusion limitation relative to a perfusion limitation is difficult with *in vivo* studies.

Nevertheless, Steinhausen *et al.* (2008) suggested that a constant *P*_vO2_ during acute warming of resting and swimming sockeye salmon was evidence for a diffusion limitation. However, *P*_vO2_ and *C*_vO2_ both decreased above *T*_opt_ in resting and maximally swimming sockeye salmon in the present study. Likewise, *P*_vO2_ and/or *C*_vO2_ decreased with warming to high temperature in resting rainbow trout, Atlantic cod, and Chinook salmon (Heath and Hughes, 1973; Sartoris *et al.*, 2003; Clark *et al.*, 2008b). Thus, a tissue diffusion limitation may not have occurred immediately, because the tissues were able to extract more oxygen from the blood at temperatures above *T*_opt_. We caution, however, that the decrease in *C*_vO2_ may not have been proportional to the increased oxygen demand with warming (Wagner, 1996). Assessments of a diffusion limitation are complicated by the decrease in 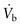, which potentially slows capillary transit time for blood, favouring oxygen diffusion. Furthermore, the decrease in blood pH accompanying anaerobic metabolism, which may facilitate oxygen unloading into the tissues via Bohr and Root effects, would provide further temporary relief from a diffusion limitation (Rummer and Brauner, 2011). All told, further research examining the possibility of a tissue diffusion limitation at warm temperature is warranted. Specifically, studies on the role of muscle morphology in limiting oxygen diffusion at high temperature in exercising fish remain a ripe area for future research.

### Limitations of the study

This study overcame the difficult challenge of swimming a sufficient number of large fish equipped with cannulae and probes to measure critical cardiorespiratory variables directly and simultaneously in order to resolve the subtle changes in the oxygen cascade when fish swim above supra-optimal temperature. Adult sockeye salmon have proved to be an effective model, because they are large enough to carry a flow probe and support repeated but strategic arterial and venous blood sampling without haemodilution. However, migrating adult sockeye salmon naturally senesce and die 4–6 weeks after they enter freshwater, which places a logistic constraint on the duration of recovery periods. Previous work has shown that *M*O_2rest_, *M*O_2max_, and aerobic scope did not differ significantly between sockeye salmon that had undergone surgery and those that had not (Eliason *et al.*, 2013), suggesting that sockeye salmon can recover quickly from surgery.

Another challenge with wild Fraser River sockeye salmon is that different populations co-migrate upstream, and individual fish are impossible to differentiate until scale and DNA analysis have been conducted several days later. Therefore, in order to ensure good statistical power, we pooled three sockeye salmon populations that all face long and difficult river migrations, have a similar *T*_opt_ for aerobic scope (∼17°C), and have a similar maximal aerobic scope, cardiac scope, and scope for heart rate (Eliason *et al.*, 2011). These three populations do not differ in *M*O_2_, 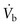, *f*_H_, or *V*_s_ at rest, when swimming, or during recovery at *T*_opt_. Also, great care was taken to create temperature groupings relative to the population-specific *T*_opt_ such that the temperature differences were only 1–3°C across the populations within a temperature grouping above *T*_opt_. As a result, the variances for *M*O_2rest_, *M*O_2max_, and aerobic scope were small for each temperature grouping (Fig. 2), which would not be the case if large population differences existed. Moreover, the Chilko population had temperature responses that paralleled those for the four temperature groupings (Figs 2 and 6).

Given the above constraints, sex-specific differences were not considered, but each temperature grouping contained approximately equal numbers of male and female sockeye salmon. Previous work (Eliason *et al.*, 2013) found no sex-specific differences in cardiorespiratory physiology and blood oxygen status at *T*_opt_ for sockeye salmon, while a study on pink salmon showed that males maintain a higher *M*O_2rest_ and achieve greater *M*O_2max_, 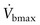, and aerobic scope (Clark *et al.*, 2011). Notably, female sockeye salmon suffer higher mortality compared with males in stressful migratory conditions, such as high temperature, elevated flow, and challenging migratory obstacles (Nadeau *et al.*, 2010; Roscoe *et al.*, 2011; Jeffries *et al.*, 2012; Martins *et al.*, 2012). Given that spawning success for a population is governed by females, this raises potential conservation concerns for any sex differences in the physiological response to temperature, which should be considered in future studies.

### Summary and conclusions

The results from the present study greatly expand upon the idea of a ‘death spiral’ for salmon swimming at supra-optimal temperatures (Farrell *et al.*, 2009), which proposes a mechanistic explanation of the failure of aerobic scope above *T*_opt_. Here we provide definitive evidence that the collapse of aerobic scope above *T*_opt_ is associated with a cardiac limitation, triggered by a collapse in scope for *f*_H_. Accordingly, a perfusion limitation for oxygen delivery to swimming muscles developed at temperatures above *T*_opt_, resulting in a mismatch between oxygen supply and demand, as evidenced by elevated lactate levels. The severity of the cardiac constraint increased with temperature to the point that elevated resting lactate, cardiac dysrhythmias, a negative scope for *f*_H_, and even post-exhaustion mortality were observed at the highest temperatures. Both *P*_aO2_ and *C*_aO2_ were unchanged with warming above *T*_opt_, suggesting no limitation in oxygen uptake at the gill with the present experimental design. With *P*_vO2_ and *C*_vO2_ (and probably blood pH, given the elevation in plasma lactate) decreasing at the highest test temperature, the possibility of a diffusion limitation also developing at the muscle remains unclear and warrants further study. We propose that a noxious venous blood environment (low pH and low *P*_vO2_) further impairs cardiac function (reducing cardiac oxygen delivery, contractility, and perhaps *f*_H_), which could initiate a positive feedback loop to exacerbate the perfusion limitation to locomotory muscle and perhaps lead to delayed mortality at extremely warm temperatures.

In terms of salmon conservation, climate change-induced increases in Fraser River temperatures have been associated with massive mortality in sockeye salmon during their spawning migration. Nevertheless, a detailed mechanistic understanding of the causes of migration failure has remained elusive. Previous work suggests that the high *en route* mortality may be partly attributed to a decrease in aerobic scope (Farrell *et al.*, 2008; Eliason *et al.*, 2011). The present study expands on this idea and suggests that salmon are unable to swim at warm temperature due to a decrease in aerobic scope caused by a cardiac limitation via insufficient scope for heart rate. While some populations of sockeye salmon have an exceptionally wide *T*_opt_ window (e.g. from 13 to 21°C for Chilko sockeye salmon; Eliason *et al.*, 2011), all populations studied to date are currently experiencing temperatures that exceed their population-specific *T*_opt_ window, and this is expected only to worsen in the future (Eliason *et al.*, 2011). The central importance of *f*_H_ in determining cardiac performance and oxygen delivery at warm temperature and the possibility of *f*_Hmax_ setting upper temperature limits may provide a useful, but simple measurement to move physiology into the field (Casselman *et al.*, 2012) and improve our knowledge of temperature tolerance of fishes as we try to conserve the natural resources that are left.

## Appendix 1

### Abbreviations

*A*–*V*_O2_, tissue oxygen extraction (*A*–*V*_O2_ = *C*_aO2_ – *C*_vO2_); *C*_aO2_, arterial oxygen content; *C*_vO2_, venous oxygen content; COT, cost of transport [COT = *M*O_2_/(*U* × 60)]; COT_net_, net cost of transport [COT_net_ = (*M*O_2_ – *M*O_2rest_)/(*U* × 60)]; , cardiovascular cost of transport ; , net cardiovascular cost of transport ; *f*_H_, heart rate; [Hb], haemaglobin concentration; Hct, haematocrit; MCHC, mean corpuscular haemaglobin concentration [MCHC = [Hb]/(Hct/100)]; *M*O_2_, rate of oxygen consumption; OCLTT, oxygen- and capacity-limited thermal tolerance; *P*_aO2_, arterial partial pressure of oxygen; *P*_vO2_, venous partial pressure of oxygen; *T*_aO2_, arterial oxygen transport ; *T*_crit_, critical temperature; *T*_min50–90_, group of fish swum at temperatures lower than *T*_opt_ at which 50–90% of maximal aerobic scope was attained; *T*_max50–90_, group of fish swum at temperatures higher than *T*_opt_ at which 50–90% of maximal aerobic scope was attained; *T*_max0–50_, group of fish swum at temperatures higher than *T*_opt_ at which 0–50% of maximal aerobic scope was attained; _opt_, optimal temperature; *T*_vO2_, venous oxygen transport ; *U*_crit_, critical swimming velocity; , cardiac output; *V*_s_, stroke volume .
